# Supplementary material for: Efficiency of Sidestream Nitritation for Modern Two-Stage Activated Sludge Plants
Source: Int J Environ Res Public Health. 2022 Oct 8;19(19):12871. doi: 10.3390/ijerph191912871 (PMC9564472; doi:10.3390/ijerph191912871)

Supplementary Material

S1: SDE Treatment Concepts

1) single-stage WWTP, SDE treatment in mainstream

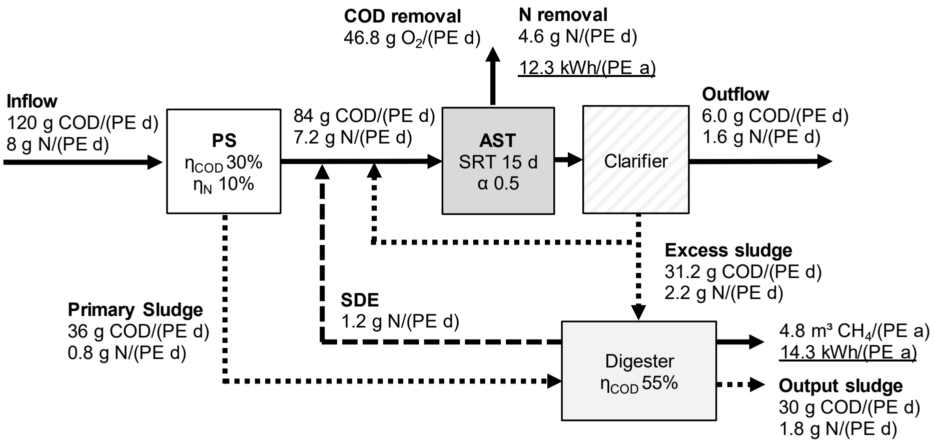

2) single-stage WWTP, SDE treatment via nitritation

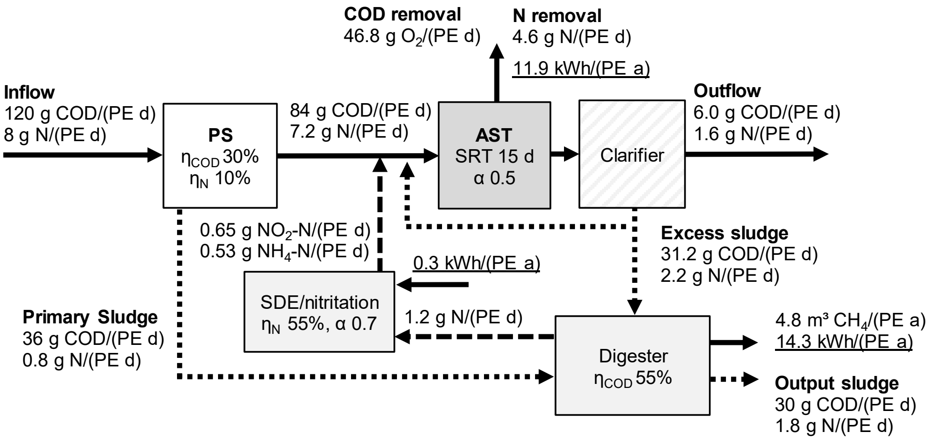

3) single-stage WWTP, SDE treatment via anammox

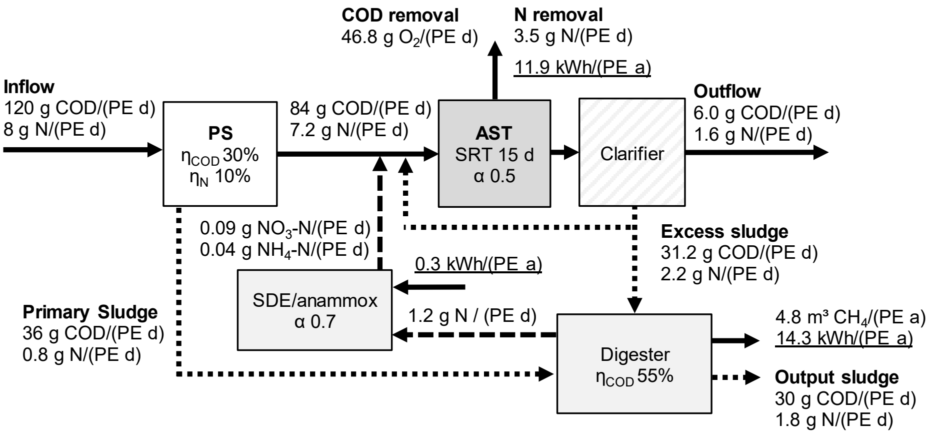

4) two-stage WWTP, SDE treatment in mainstream

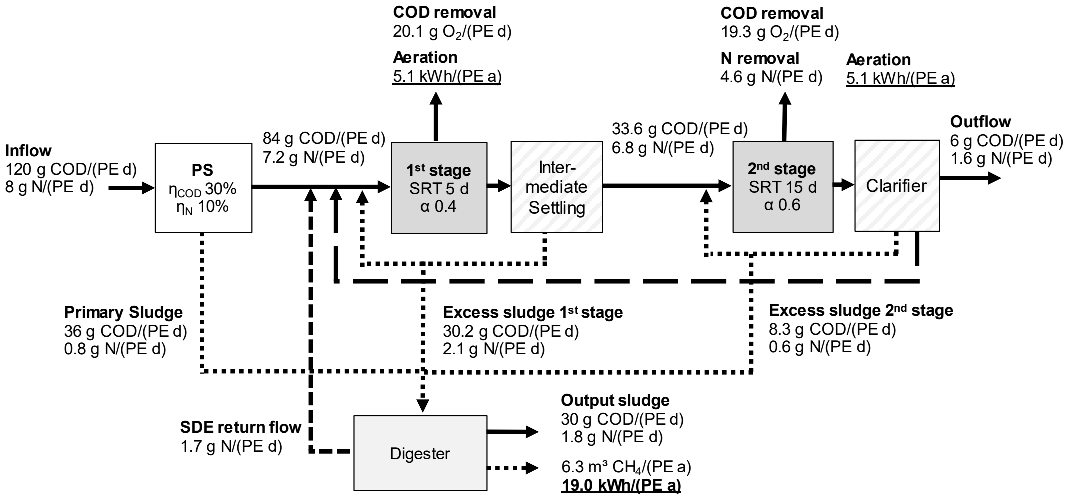

5) two-stage WWTP, SDE treatment via nitritation

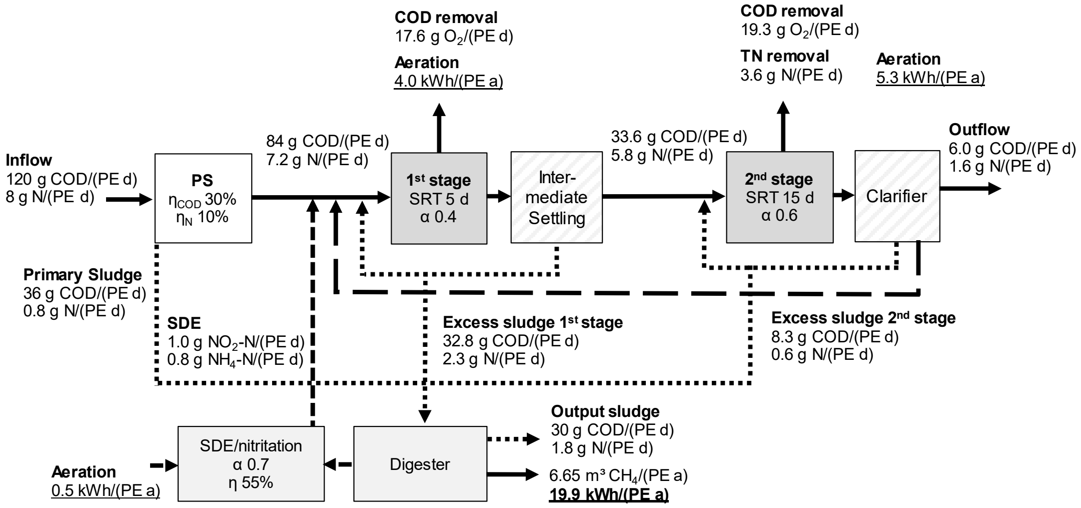

6) two-stage WWTP, SDE treatment via anammox

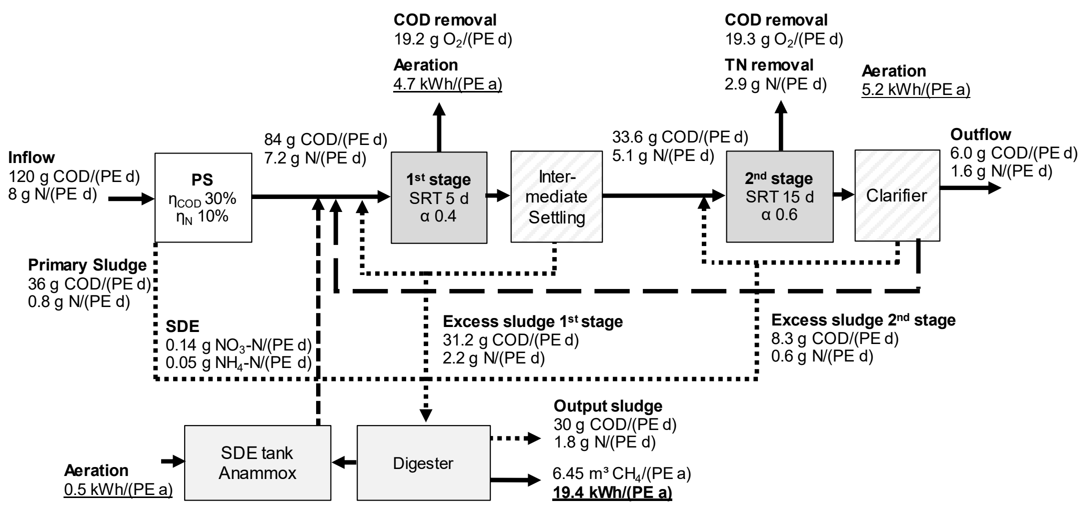

Supplement: Supplementary file 1 [file ijerph-19-12871-s001.zip › ijerph-1868840-supplementary.pdf]
